# Polypyridine Os^II^ complexes electrochemical data

**DOI:** 10.1016/j.dib.2020.106454

**Published:** 2020-10-22

**Authors:** Deidré van der Westhuizen, Ayyavoo Kannan, Jeanet Conradie, Karel G. von Eschwege

**Affiliations:** Department of Chemistry, University of the Free State, PO Box 339, Bloemfontein 9300, South Africa

**Keywords:** Osmium, Bipyridyl, Phenanthroline, Photocatalyst, Dye, Redox prediction

## Abstract

The data presented in this article are relevant to the research article, “Electrochemistry of Os bipyridyl and phenanthroline complexes, comparison with Ru and Fe” (van der Westhuizen, 2020). Cyclic voltammograms illustrating Os^II/III^ oxidations of eight osmium(II) complexes are presented in this article. The data were obtained under similar experimental conditions, at scan rates with magnitudes ranging from 0.05 V.s^−1^ to 10.00 V.s^−1^, in acetonitrile as solvent and tetrabutylammonium hexafluorophosphate as supporting electrolyte. Potentials are reported versus the iron(II) redox couple in ferrocene.

## Specifications Table

**Table:** 

Subject	Chemistry
Specific subject area	Electrochemistry
Type of data	Table, text file, graph, figure
How data were acquired	PARTAT 2273, Advanced Electrochemical System
Data format	Raw and Analyzed
Parameters for data collection	CV measurements were done at 293 K. Synthesized samples were used. Degassed the solvent-electrolyte solution, in this case acetonitrile, in the electrochemical cell with Ar(g) for approximately 10 min. Sample addition to the acetonitrile-electrolyte solution and degassed for approximately 3 min. A blanket of Ar(g) was maintained in the cell for the duration of the electrochemical analysis.
Description of data collection	Electrochemical analyses of all the samples were done in an electrochemical cell (2 mL), containing a glassy carbon working electrode, a Pt pseudo reference electrode and a Pt auxiliary electrode. The electrochemical cell was connected to a BAS 100 B/W and electrochemical analyzer, and the obtained data were transferred to Excel for data analysis and diagram preparation.
Data source location	Department of Chemistry, University of the Free State: Bloemfontein: South Africa: 29°06′36.1″S 26°11′09.2″E
Data accessibility	Primary data available as Supplementary Information
Related research article	K.G. von Eschwege, J. Conradie, D. van der Westhuizen, Electrochemistry of Os bipyridyl and phenanthroline complexes, comparison with Ru and Fe, Electroanalysis (2020) in press. https://doi.org/10.1002/elan.202060300

## Value of the Data

• The electrochemical data illustrate the influence of different polypyridine ligand functional groups on the ease of Os^II/III^ oxidation, at scan rates 0.05 – 10.0 V s^−1^.
• The data is relevant to research and development of redox indicators, and electro- or photocatalysts in dye-sensitized solar cell or solar liquid fuels manufacturing, etc.
• The range of nine cyclic voltammetry scan rates for each of the eight compounds provide data sets from which best methods and derivatives may be selected in future experiments.

## 1 Data Description

**Fig. 1:**
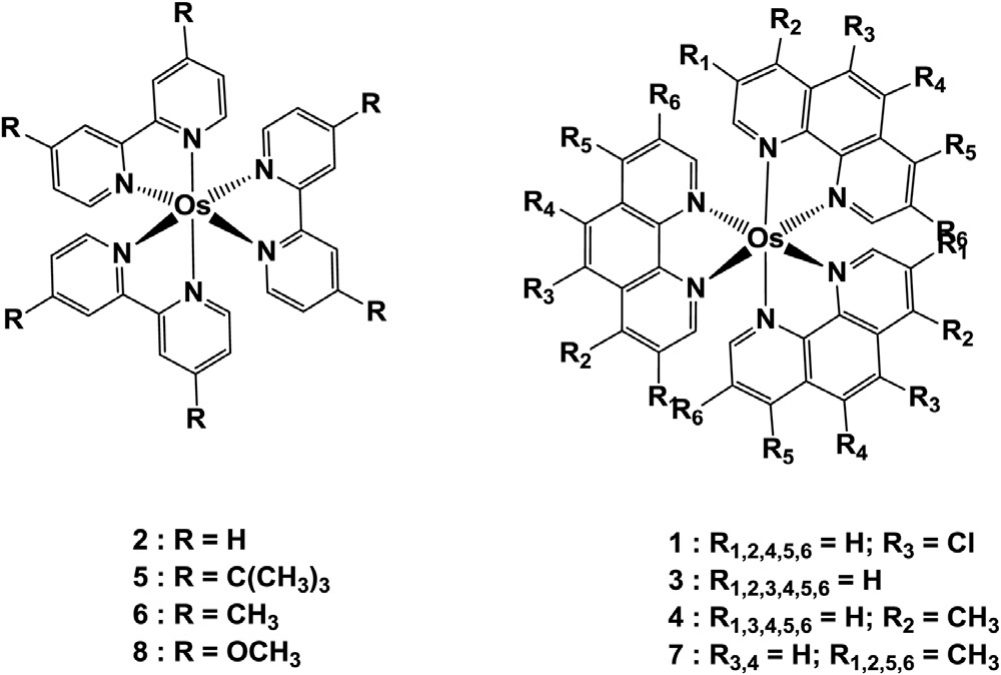
Complex numbering and structure of Os^II^ polypyridine complexes.

**Fig. 2:**
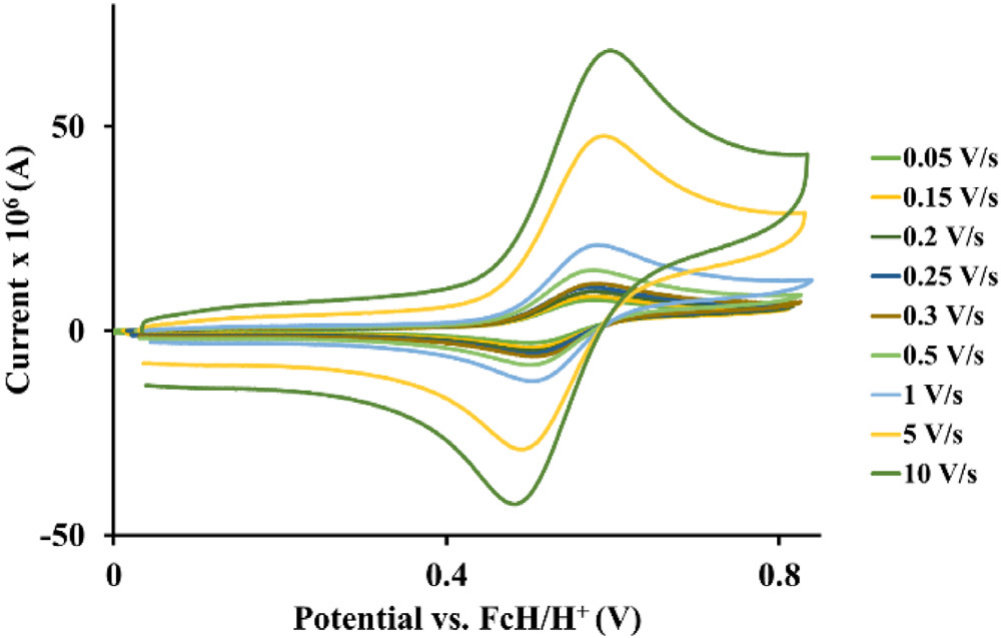
Cyclic voltammograms of *tris*(5‑chloro-1,10-phenanthroline)osmium *bis*(tetrafluoroborate), 1, at scan rates 0.05 V/s to 10 V/s in the positive direction.

**Fig. 3:**
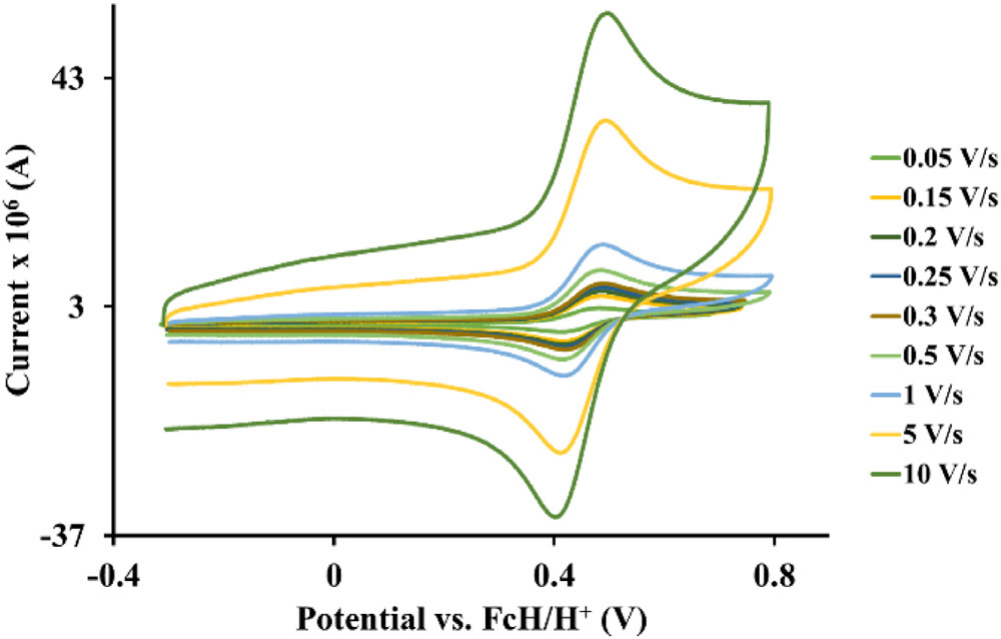
Cyclic voltammograms of *tris*(2,2′-bipyridine)osmium *bis*(tetrafluoroborate), 2, at scan rates 0.05 V/s to 10 V/s in the positive direction.

**Fig. 4:**
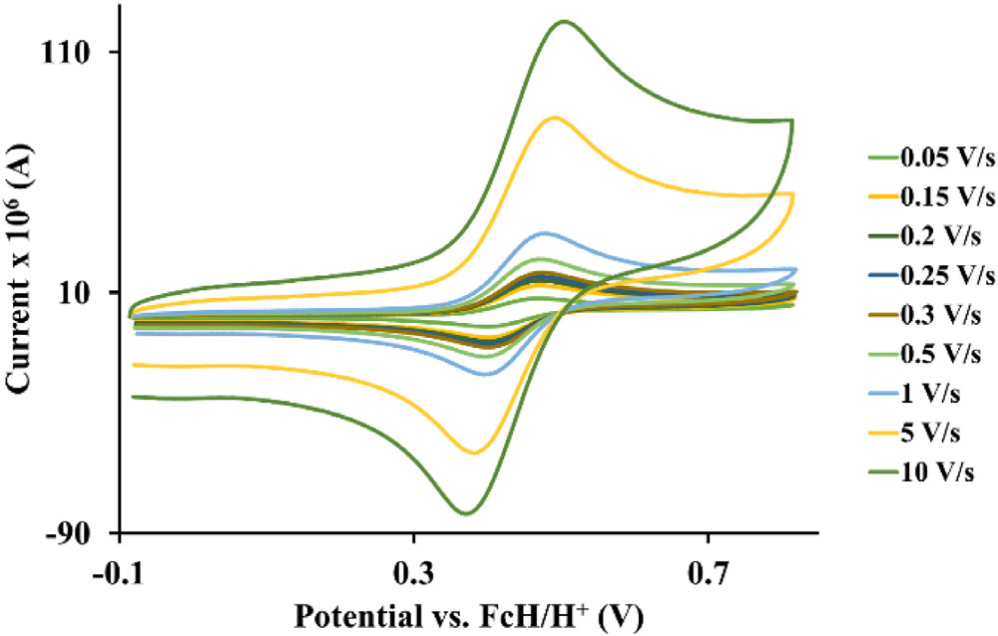
Cyclic voltammograms of *tris*(1,10-phenanthroline)osmium *bis*(tetrafluoroborate), 3, at scan rates 0.05 V/s to 10 V/s in the positive direction.

**Fig. 5:**
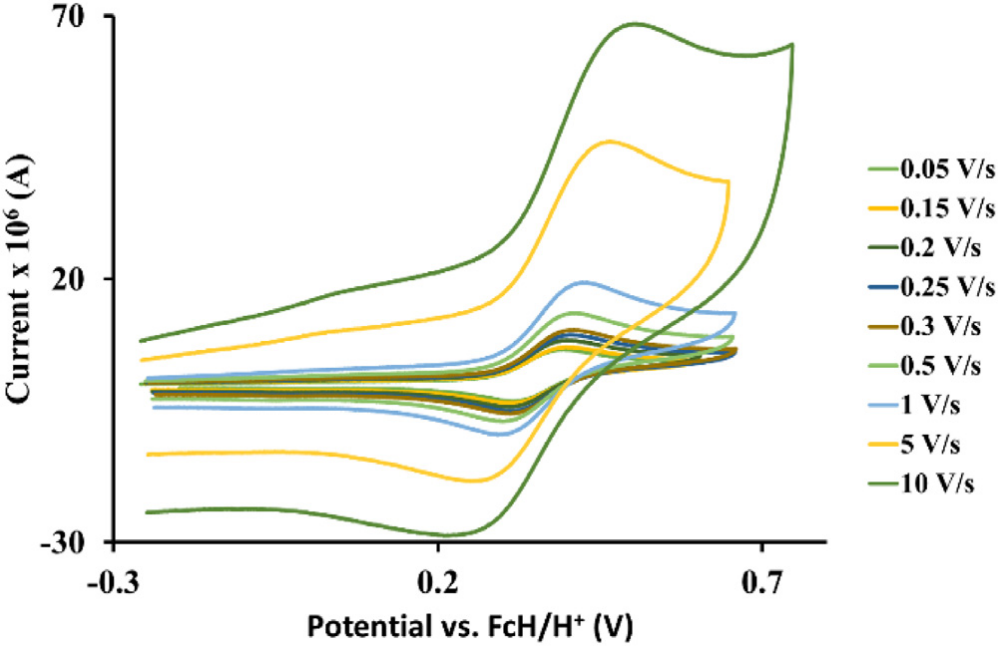
Cyclic voltammograms of *tris*(4-methyl-1,10-phenanthroline)osmium *bis*(tetrafluoroborate), 4, at scan rates 0.05 V/s to 10 V/s in the positive direction.

**Fig. 6:**
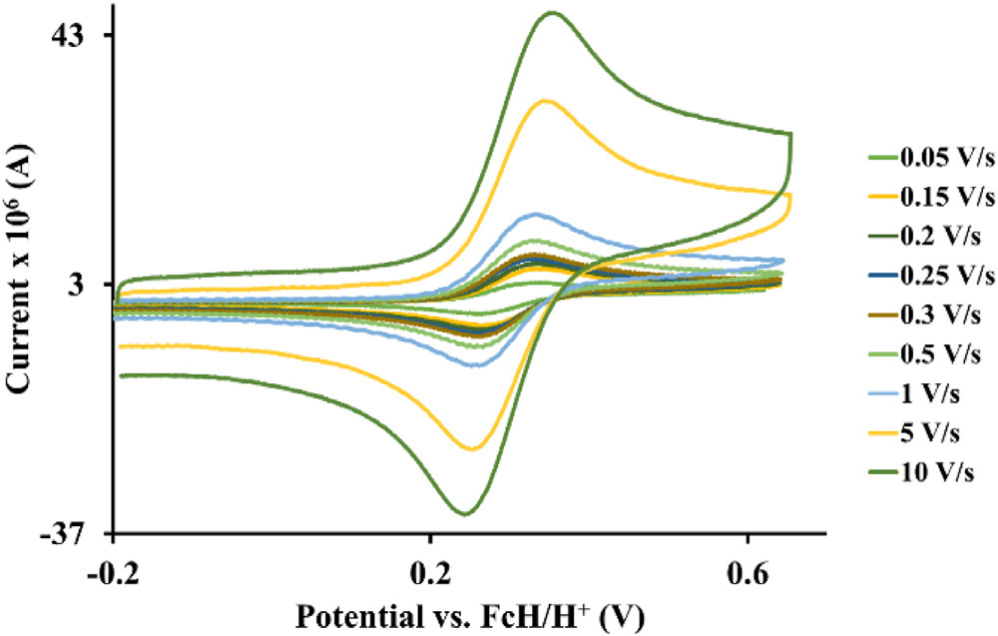
Cyclic voltammograms of *tris*(4,4′-di‑*tert*‑butyl‑2,2′-bipyridine)osmium *bis*(tetrafluoroborate), 5, at scan rates 0.05 V/s to 10 V/s in the positive direction.

**Fig. 7:**
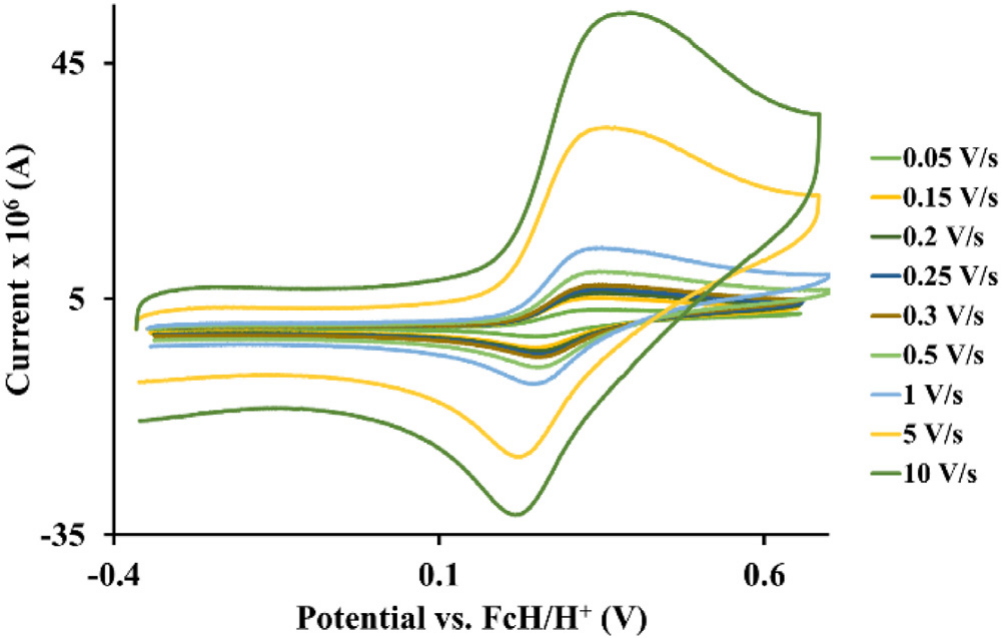
Cyclic voltammograms of *tris*(4,4′-dimethyl-2,2′-bipyridine)osmium *bis*(tetrafluoroborate), 6, at scan rates 0.05 V/s to 10 V/s in the positive direction.

**Fig. 8:**
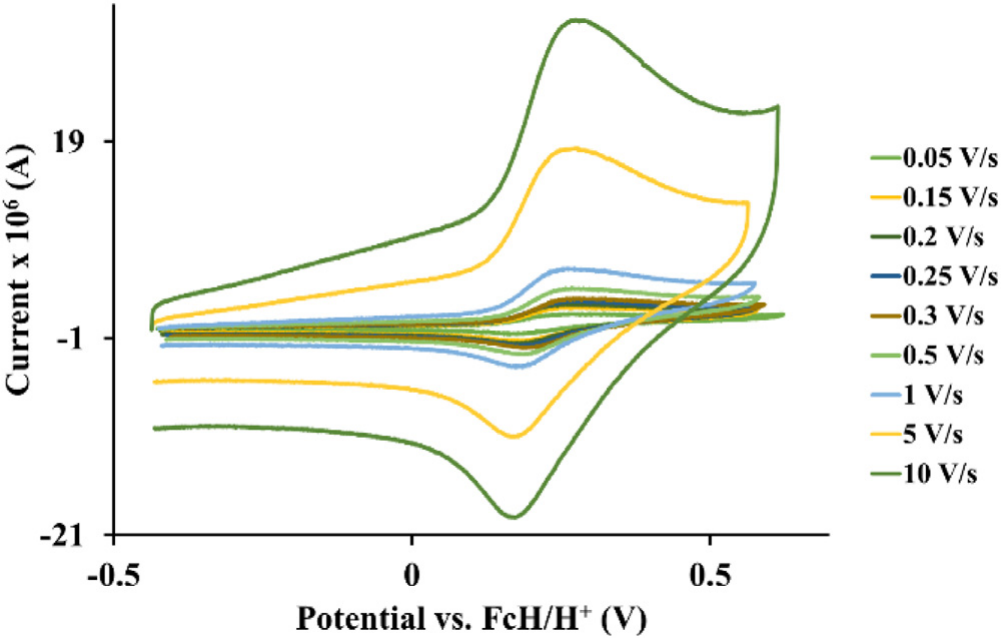
Cyclic voltammograms of *tris*(3,4,7,8-tetramethyl −1,10-phenanthroline)osmium *bis*(tetrafluoroborate), 7, at scan rates 0.05 V/s to 10 V/s in the positive direction.

**Fig. 9:**
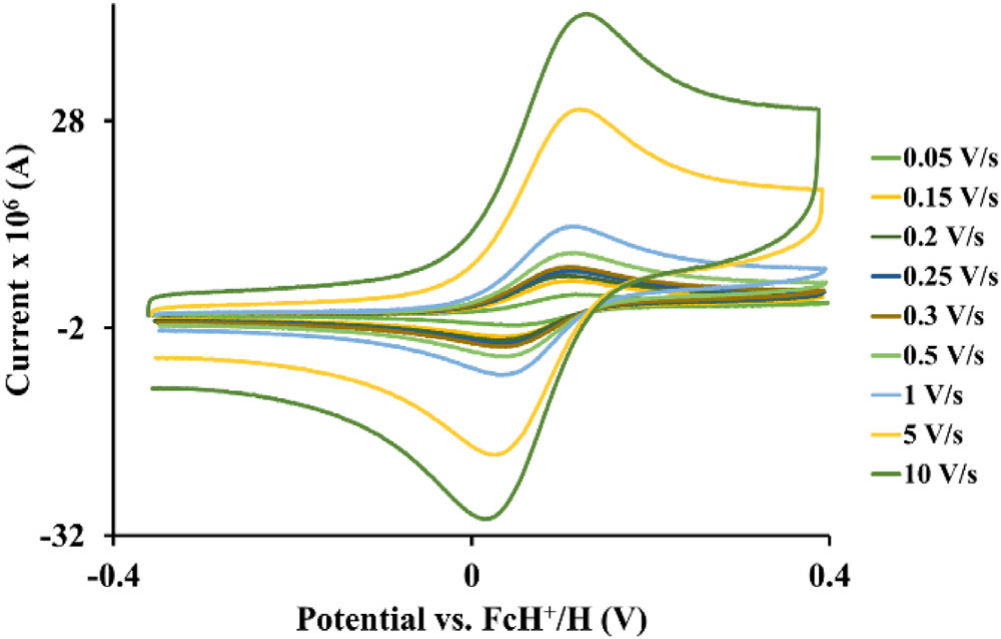
Cyclic voltammograms of *tris*(4,4′-dimethoxy-2,2′-bipyridine)osmium *bis*(tetrafluoroborate), 8, at scan rates 0.05 V/s to 10 V/s in the positive direction.

The redox data of eight octahedral Os^II^ complexes are presented in this article. These complexes, **1**–**8**, contain different polypyridine ligands, namely, bipyridine-, substituted bipyridine-, phenanthroline- and substituted phenanthroline ligands, see Fig 1. The data presented in this study are related to the research article [1] where cyclic voltammograms (CVs) and peak voltage data obtained at 0.10 Vs^−1^ scan rates are reported and correlated with DFT computed descriptors. The redox data obtained for the Os^II^ complexes, containing different polypyridine ligands with different electron donating properties [2], are useful in applications of redox indicators, catalysts and photo-active mediators in dye sensitized solar cells (DSSC) [3,[4], [5]. Electrochemical data obtained from CVs at scan rates 0.05 Vs^−1^ – 10.00 Vs^−1^, see Figs. 2–9, are tabulated in Tables 1–8. All related primary data are available in the supplementary data file.

**Table 1:** *Tris*(5‑chloro-1,10-phenanthroline)osmium *bis*(perchlorate), 1, electrochemical data (potential in V vs. FcH/FcH^+^) of the Os^II/III^ redox couple in acetonitrile (CH_3_CN) for ca 0.005 mol.dm^−3^ complex solution at the indicated scan rates.

Scan Rate (V/s)	*E_pa_* (V)	10^6^*I_pa_* (A)	*E_pc_* (V)	10^6^*I_pc_* (A)	E°’ (V)	Δ*E* (V)	*I_p_*_c_/*I_pa_*
0.05	0.572	3.13	0.498	3.20	0.535	0.074	0.98
0.15	0.573	5.99	0.497	6.13	0.535	0.076	0.98
0.20	0.571	6.63	0.499	6.75	0.535	0.072	0.98
0.25	0.574	7.50	0.497	7.68	0.535	0.077	0.98
0.30	0.577	7.99	0.494	8.17	0.535	0.083	0.98
0.50	0.574	11.46	0.496	11.72	0.535	0.078	0.98
1.00	0.575	14.02	0.495	14.37	0.535	0.080	0.98
5.00	0.585	32.69	0.486	33.46	0.535	0.099	0.98
10.0	0.592	45.56	0.479	46.44	0.535	0.113	0.98

**Table 2:** *Tris*(2,2′-bipyridine)osmium *bis*(tetrafluoroborate), 2, electrochemical data (potential in V vs. FcH/FcH^+^) of the Os^II/III^ redox couple in acetonitrile (CH_3_CN) for ca 0.005 mol.dm^−3^ complex solution at the indicated scan rates.

Scan Rate (V/s)	*E_pa_* (V)	10^6^*I_pa_* (A)	*E_pc_* (V)	10^6^*I_pc_* (A)	*E*°’ (V)	Δ*E* (V)	*I_p_*_c_/*I_pa_*
0.05	0.490	1.87	0.415	1.90	0.452	0.075	0.98
0.15	0.486	3.53	0.418	3.60	0.452	0.068	0.98
0.20	0.485	4.16	0.419	4.25	0.452	0.066	0.98
0.25	0.485	4.76	0.419	4.85	0.452	0.066	0.98
0.30	0.485	5.27	0.419	5.40	0.452	0.066	0.98
0.50	0.486	6.55	0.419	6.71	0.452	0.067	0.98
1.00	0.486	9.96	0.419	10.12	0.452	0.067	0.98
5.00	0.494	22.16	0.410	22.58	0.452	0.084	0.98
10.0	0.501	32.89	0.403	33.65	0.452	0.098	0.98

**Table 3:** *Tris*(1,10-phenanthroline)osmium *bis*(tetrafluoroborate), 3, electrochemical data (potential in V vs. FcH/FcH^+^) of the Os^II/III^ redox couple in acetonitrile (CH_3_CN) for ca 0.005 mol.dm^−3^ complex solution at the indicated scan rates.

Scan Rate (V/s)	*E_pa_* (V)	10^6^*I_pa_* (A)	*E_pc_* (V)	10^6^*I_pc_* (A)	*E*°’ (V)	Δ*E* (V)	*I_pc_*/*I_pa_*
0.05	0.479	5.15	0.409	5.25	0.444	0.070	0.98
0.15	0.478	10.34	0.411	10.59	0.444	0.067	0.98
0.20	0.481	12.09	0.408	12.36	0.444	0.073	0.98
0.25	0.480	13.16	0.408	13.47	0.444	0.072	0.98
0.30	0.481	14.41	0.408	14.71	0.444	0.073	0.98
0.50	0.484	19.01	0.404	19.42	0.444	0.080	0.98
1.00	0.486	25.64	0.402	26.26	0.444	0.084	0.98
5.00	0.499	54.95	0.389	56.26	0.444	0.110	0.98
10.0	0.512	80.50	0.376	81.93	0.444	0.136	0.98

**Table 4:** *Tris*(4-methyl-1,10-phenanthroline)osmium *bis*(tetrafluoroborate), 4, electrochemical data (potential in V vs. FcH/FcH^+^) of the Os^II/III^ redox couple in acetonitrile (CH_3_CN) for ca 0.005 mol.dm^−3^ complex solution at the indicated scan rates.

Scan Rate (V/s)	*E_pa_* (V)	10^6^*I_pa_* (A)	*E_pc_* (V)	10^6^*I_pc_* (A)	*E*°’ (V)	Δ*E* (V)	*I_pc_*/*I_pa_*
0.05	0.391	4.13	0.322	4.23	0.356	0.069	0.98
0.15	0.398	4.99	0.314	5.11	0.356	0.084	0.98
0.20	0.403	5.36	0.310	5.48	0.356	0.093	0.98
0.25	0.401	5.99	0.312	6.12	0.356	0.089	0.98
0.30	0.403	6.32	0.309	6.48	0.356	0.094	0.98
0.50	0.414	8.49	0.299	8.67	0.356	0.115	0.98
1.00	0.421	10.33	0.290	10.58	0.356	0.131	0.98
5.00	0.464	19.57	0.249	19.90	0.356	0.215	0.98
10.0	0.496	28.17	0.217	28.65	0.356	0.279	0.98

**Table 5:** *Tris*(4,4′-di‑*tert*‑butyl‑2,2′-bipyridine)osmium *bis*(tetrafluoroborate), 5, electrochemical data (potential in V vs. FcH/FcH^+^) of the Os^II/III^ redox couple in acetonitrile (CH_3_CN) for ca 0.005 mol.dm^−3^ complex solution at the indicated scan rates.

Scan Rate (V/s)	*E_pa_* (V)	10^6^*I_pa_* (A)	*E_pc_* (V)	10^6^*I_pc_* (A)	*E*°’ (V)	Δ*E* (V)	*I_pc_*/*I_pa_*
0.05	0.338	2.35	0.259	2.40	0.298	0.079	0.98
0.15	0.331	4.55	0.266	4.63	0.298	0.065	0.98
0.20	0.336	5.49	0.260	5.59	0.298	0.076	0.98
0.25	0.335	5.87	0.261	5.97	0.298	0.074	0.98
0.30	0.332	6.34	0.264	6.46	0.298	0.068	0.98
0.50	0.329	8.03	0.267	8.18	0.298	0.062	0.98
1.00	0.333	11.83	0.262	12.06	0.298	0.071	0.98
5.00	0.343	25.41	0.254	26.06	0.298	0.089	0.98
10.0	0.353	32.62	0.243	33.40	0.298	0.110	0.98

**Table 6:** *Tris*(4,4′-methyl-2.2′-bipyridine)osmium *bis*(tetrafluoroborate), 6, electrochemical data (potential in V vs. FcH/FcH^+^) of the Os^II/III^ redox couple in acetonitrile (CH_3_CN) for ca 0.005 mol.dm^−3^ complex solution at the indicated scan rates.

Scan Rate (V/s)	*E_pa_* (V)	10^6^*I_pa_* (A)	*E_pc_* (V)	10^6^*I_pc_* (A)	*E*°’ (V)	Δ*E* (V)	*I_p_*_c_/*I_pa_*
0.05	0.335	2.21	0.237	2.27	0.286	0.098	0.98
0.15	0.324	4.39	0.247	4.46	0.286	0.077	0.98
0.20	0.333	4.56	0.239	4.68	0.286	0.094	0.98
0.25	0.335	5.32	0.237	5.45	0.286	0.098	0.98
0.30	0.334	5.78	0.238	5.92	0.286	0.096	0.98
0.50	0.335	7.49	0.236	7.62	0.286	0.099	0.98
1.00	0.339	11.23	0.232	11.50	0.286	0.107	0.98
5.00	0.351	20.99	0.221	21.52	0.286	0.130	0.98
10.0	0.365	31.24	0.206	31.90	0.286	0.159	0.98

**Table 7:** *Tris*(3,4,7,8-tetramethyl-1,10-phenanthroline)osmium *bis*(tetrafluoroborate), 7, electrochemical data (potential in V vs. FcH/FcH^+^) of the Os^II/III^ redox couple in acetonitrile (CH_3_CN) for ca 0.005 mol.dm^−3^ complex solution at the indicated scan rates.

Scan Rate (V/s)	*E_pa_* (V)	10^6^*I_pa_* (A)	*E_pc_* (V)	10^6^*I_pc_* (A)	*E*°’ (V)	Δ*E* (V)	*I_pc_*/*I_pa_*
0.05	0.263	0.79	0.164	0.82	0.213	0.099	0.97
0.15	0.254	1.58	0.173	1.61	0.213	0.081	0.98
0.20	0.260	1.76	0.166	1.80	0.213	0.094	0.97
0.25	0.258	1.97	0.169	2.06	0.213	0.089	0.96
0.30	0.260	2.24	0.167	2.37	0.213	0.093	0.95
0.50	0.254	2.69	0.173	2.82	0.213	0.081	0.95
1.00	0.262	3.10	0.164	3.25	0.213	0.098	0.96
5.00	0.268	10.37	0.159	10.60	0.213	0.109	0.98
10.0	0.265	17.39	0.162	17.99	0.213	0.103	0.97

**Table 8:** *Tris*(4,4′-dimethoxy-2,2′bipyridine)osmium *bis*(tetrafluoroborate), 8, electrochemical data (potential in V vs. FcH/FcH^+^) of the Os^II/III^ redox couple in acetonitrile (CH_3_CN) for ca 0.005 mol.dm^−3^ complex solution at the indicated scan rates.

Scan Rate (V/s)	*E_pa_* (V)	10^6^ I_p_*_a_* (A)	*E_pc_* (V)	10^6^*I_pc_* (A)	*E*°’ (V)	Δ*E* (V)	*I_pc_*/*I_pa_*
0.05	0.113	2.10	0.036	2.14	0.074	0.077	0.98
0.15	0.114	3.70	0.035	3.77	0.074	0.079	0.98
0.20	0.112	4.26	0.036	4.37	0.074	0.076	0.98
0.25	0.110	4.79	0.039	4.90	0.074	0.071	0.98
0.30	0.110	5.43	0.039	5.54	0.074	0.071	0.98
0.50	0.113	6.94	0.036	7.09	0.074	0.077	0.98
1.00	0.113	10.57	0.035	10.79	0.074	0.078	0.98
5.00	0.121	19.98	0.027	20.32	0.074	0.094	0.98
10.0	0.132	28.20	0.016	28.65	0.074	0.116	0.98

## 2 Experimental Design, Materials and Methods

The experimental setup is the same as described in our previous articles [6,7], i.e. electrochemical studies utilizing cyclic voltammetric measurements were done on an Advanced Electrochemical System with personal computer, utilizing PARSTAT 2273 Powersuite software. Measurements were done at 293 K. Consecutive experiments under similar experimental conditions illustrated that all the formal oxidation potentials could be duplicated within 0.005 V and were not influenced by scan rate. CV data presented in this article are obtained from single CVs at different scan rates as indicated in the tables and figures. Cyclic voltammetric measurements were performed on 0.005 mol dm^−3^ solutions of the complex, dissolved in CH_3_CN, containing 0.200 mol dm^−3^ tetrabutylammonium hexafluorophosphate (TBAPF_6_, [NBu_4_][PF_6_]) as supporting electrolyte. Measurements were conducted under a blanket of purified Argon. A three-electrode cell consisting of a Pt auxiliary electrode, a glassy carbon (surface area 3.14 × 10^−6^ m^2^) working electrode and a Pt-wire pseudo reference electrode were used. The working electrode was polished on a Buhler polishing mat; first with 1 µm and lastly with 0.25 µm diamond paste. Scan rates were between 0.05 and 10.00 V.*s* ^−^ ^1^. All experimental potentials were referenced against the redox couple of ferrocene FcH/FcH^+^ (IUPAC) [8].

## CRediT Author Statement

**Deidré van der Westhuizen:** Synthesis, Experimental measurements, Writing. **Ayyavoo Kannan:** Synthesis. **Jeanet Conradie:** Supervision, Editing. **Karel von Eschwege:** Conceptualization, Supervision, Editing.

## Declaration of Competing Interest

The authors declare that they have no known competing financial interests or personal relationships which have or could be perceived to have influenced the work reported in this article.

## Appendix Supplementary materials

Image, application 1
